# Soil texture and depth differentially regulate total and available nutrient stoichiometry in Yilong Lake basin agroecosystem, China

**DOI:** 10.1371/journal.pone.0361813

**Published:** 2026-10-29

**Authors:** Yongru Lai, Luoqi Zhao, Chenyang Zhang, Zijun Du, Yandan Wang, Tianyi Pu, Change Liu, Changqun Duan

**Affiliations:** 1 Yunnan Key Laboratory for Plateau Mountain Ecology and Restoration of Degraded Environments, Yunnan University, Kunming, Yunnan, China; 2 Central Yunnan Field Scientific Station for Restoration of Ecological Function & Yunnan International Joint Research Center of Plateau Lake Ecological Restoration and Watershed Management, Yunnan Think Tank for Ecological Civilization Construction, Yunnan University, Kunming, China; 3 Southwestern United Graduate School & Institute of International Rivers and Eco-security, Yunnan University, Kunming, China; The University of Hong Kong, HONG KONG

## Abstract

The stoichiometric ratios of soil available nutrients serve as a pivotal indicator for assessing the sustainability of farmland and nutrient cycling efficiency within fragile ecosystems. Soil texture and depth are significant factors influencing nutrient availability, however, their regulatory mechanisms within highland lakeshore agricultural systems remain poorly understood. This study, conducted in the Yilong Lake watershed of the low-latitude plateau in southwest China, collected 216 soil samples. It innovatively determined both total organic carbon, nitrogen, and phosphorus (SOC, TN, TP) and available carbon, nitrogen, and phosphorus (DOC, AN, AP) simultaneously. Path analysis was employed to examine the direct and indirect associations between soil texture (Clay, Loam, Clay Loam, Sand) and depth (0–20 cm, 20–40 cm, 40–60 cm) on nutrient ratio measurements. Findings indicate: (1) Distinct nutrient limitation and leaching risk patterns emerged across soil textures: Loam exhibited strongest carbon and nitrogen sequestration capacity; Although Clay Loam exhibits reduced phosphorus availability due to iron and aluminium oxide immobilisation, it demonstrates pronounced nitrogen limitation rather than phosphorus limitation; Sand though rich in available phosphorus, had the lowest C:N, suggesting heightened carbon and nitrogen leaching risks. (2) Soil depth significantly altered nutrient ratios: topsoil (0–20 cm) showed marked nutrient enrichment, while subsoil (40–60 cm) exhibited elevated TN:TP, suggesting that nitrification may have intensified with increasing depth. (3) Path analysis revealed that texture directly regulates nutrient measurement ratios (DOC:AN) and indirectly governs C:N:P balance by influencing SOC, AP, and AN content; depth directly or indirectly affects measurement relationships through pathways such as TP. This study elucidates the key regulatory roles of soil texture and depth in nutrient ratios, particularly available nutrient balance, providing direct scientific evidence for implementing differentiated, precision nutrient management based on soil physical properties in Yilong Lake basin agroecosystems and other ecologically fragile areas.

## 1 Introduction

The development of sustainable agriculture confronts challenges encompassing food security, environmental governance, and climate adaptation. In ecologically fragile regions such as plateau lake basins, intensive agricultural production safeguards food supply yet introduces significant nitrogen and phosphorus non-point source pollution risks, threatening downstream water safety and ecosystem health [1,2]. Soil carbon (C), nitrogen (N) and phosphorus (P) stoichiometry is pivotal for assessing farmland sustainability and nutrient cycling efficiency in these ecosystems [3,4]. However, traditional research has predominantly focused on total organic carbon, total nitrogen, and total phosphorus (SOC, TN, TP), which reflect long-term nutrient stocks but inadequately capture the immediate bioavailability driving short-term biogeochemical cycles [5,6]. In contrast, the stoichiometric ratios of available nutrients—dissolved organic carbon (DOC), ammonium and nitrate nitrogen (AN) and available phosphorus (AP)—directly influence microbial metabolic activities, instantaneous crop uptake, and environmental loss potential via leaching or runoff [7,8]. Recent advances demonstrate that microbial resource limitation and carbon use efficiency are tightly coupled with the stoichiometry of available C, P and N, rather than total nutrient pools alone [9,10]. Despite this significance, systematic investigations into the drivers of available nutrient stoichiometry remain underdeveloped, particularly in agroecosystems where physical soil properties fundamentally regulate nutrient bioavailability [11].

Soil texture is hypothesized to serve as a primary physical template governing the sequestration, transformation, and migration of available nutrients through distinct mechanisms. Clay-rich soils, characterized by high specific surface area and abundant Fe/Al oxyhydroxides, exhibit strong chemical sorption of phosphate, potentially reducing AP availability despite high TP stocks [12,13]; concurrently, their micropore structure may enhance physicochemical stabilization of organic matter, retarding nitrogen mineralization [14,15]. Conversely, Sand with coarse porosity and limited sorption sites promotes rapid water infiltration and nutrient leaching, often depleting SOC and TN [16,17]. Loam, possessing intermediate particle-size distributions, is generally expected to maintain superior water-holding capacity and nutrient retention [18,19]. Nevertheless, a persistent debate exists regarding whether texture controls nutrient dynamics primarily through physical protection of organic matter or through chemical sorption on mineral surfaces, leading to divergent predictions for labile nutrient stoichiometry [20,21]. Consequently, while texture effects on total nutrient stocks or single available elements have been documented, the integrated stoichiometric balance of labile fractions (DOC:AN, AN:AP, DOC:AP) across contrasting textures, and the mechanisms driving divergent patterns, remain insufficiently understood.

Soil depth represents a second fundamental dimension structuring nutrient availability through vertical gradients in root distribution, organic matter input, microbial activity, and weathering-deposition processes. In natural ecosystems, declining C:P and N:P with depth reflect phosphorus limitation in subsurface horizons [22,23]. However, the vertical patterns of available nutrient stoichiometry in cultivated ecosystems may diverge from natural systems because tillage and irrigation homogenize upper profiles while fertiliser application creates anthropogenic enrichment gradients [6,24]. Importantly, the interactive effects of soil texture and depth on available nutrient stoichiometry have rarely been examined in tandem. While texture determines the intrinsic capacity for nutrient retention and release, depth modulates the intensity of biological and hydrological drivers; their combination forms a three-dimensional framework that may generate non-additive effects on nutrient bioavailability and loss potential [25,26]. Whether and how these two physical dimensions jointly regulate the balance of available C, N, and P in agroecosystems remains an open question.

The Yilong Lake watershed, situated on the low-latitude plateau of southwestern China, serves as an ideal natural laboratory for addressing these gaps. This watershed is characterized by intensive cultivation of melons, fruits, vegetables and grains, diverse soil texture types (Clay, Loam, Clay Loam, and Sand), seasonally concentrated rainfall, and the ecological fragility of shallow lakes [27,28]. Specifically, this unique setting creates a tightly coupled hydrological–soil interface wherein (1) a high evaporation-to-precipitation ratio (1,908 mm yr^–1^ vs. 920 mm yr^–1^) concentrates surface nutrients and promotes upward capillary movement of dissolved P [28]; (2) seasonally concentrated monsoon rainfall (~80% falling between May and October) generates intense surface runoff and interflow that transports labile nutrients from farmland to the lake [28,29]; and (3) shallow groundwater tables (0.2–2.3 m in the dry season) facilitate vertical nutrient migration via capillary rise [30]. These coupled hydrological–soil processes are amplified by intensive fertilization, creating a high-risk corridor for non-point source pollution. Within this framework, soil texture and depth serve as the fundamental physical templates regulating water retention, infiltration, and nutrient sorption, thereby modulating the source strength of agricultural runoff. Despite these high-risk conditions, systematic understanding is still lacking regarding how available nutrient ratios are controlled by the interaction of soil texture and depth in plateau lake-marginal agricultural ecosystems.

To address these gaps, this study focuses on farmland within the Yilong Lake basin to elucidate the regulatory mechanisms by which soil texture and depth influence available nutrient stoichiometry. We propose three testable hypotheses: (1) Soil texture exerts a stronger control over the stocks and stoichiometric ratios of total C, P and N (SOC:TN, SOC:TP, TN:TP) than soil depth. (2) Compared to total nutrients, the stoichiometric ratios of available nutrients (DOC:AN, AN:AP, DOC:AP) are more significantly influenced by the interaction between soil texture and depth, because labile fractions are simultaneously regulated by physical retention capacity (texture) and vertical biogeochemical gradients (depth) (3) Soil texture primarily exerts indirect control over the stoichiometric balance of available nutrients and their potential loss risk, chiefly through regulating key soil attributes such as SOC content and pH, whereas depth directly alters nutrient ratios by affecting TP dynamics and mineralization intensity. By validating these hypotheses, this study aims to establish differentiated, precision nutrient management strategies based on soil physical properties for the Yilong Lake basin agroecosystem and other ecologically fragile areas, providing a robust scientific basis.

## 2 Materials and methods

### 2.1 Site description

Yilong Lake (102°30′–102°38′E, 23°39′–23°42′N) is a shallow freshwater lake (mean depth 2.75 m, area 31 km^2^) in China’s Yunnan-Guizhou Plateau, and most of the inhabitants around the lake make their living by cultivating crops [28]. Characterized by a subtropical monsoon climate (annual temperature 18°C, precipitation 920 mm, evaporation 1,908 mm), the region exhibits: Surface runoff and rainfall account for 77% of inflow [31], amplifying nutrient transport from farmlands to the lake. High evaporation-to-precipitation ratio (2.1:1) exacerbates soil salinization risks, while seasonal rainfall variability threatens crop yields. Intensive fertiliser application and shallow lake depths lead to frequent algal outbreaks, endangering aquatic biodiversity and water security. This confluence of agricultural intensity, hydrological dynamics, and climate variability makes Yilong Lake basin a paradigmatic site for studying sustainable resource management at the food-water-climate interface.

### 2.2 Experimental design and sampling

Field tests were undertaken in the farmland around Yilong Lake at the locations shown in Fig 1a. Field access and soil sampling were conducted with the permission of the local village committees and individual farmland contract-holders in the Yilong Lake basin. Because sampling was restricted to privately managed, non-protected agricultural land and did not involve any endangered or protected species, no additional administrative or ethical permits were required. Soils from a representative agricultural field were selected, and a laser particle size analyser was employed to ascertain the soil particle size distribution. The soil textures in the main cultivated lands of the Yilong Lake basin were classified as Clay, Loam, Clay Loam, and Sand according to the International Classification System (Fig 1b). For each soil texture, three geographically independent sites were selected, with a minimum distance of 20 metres between any two sites, to ensure spatial independence. Vegetable, grain, and fruit crops were represented in each soil-texture sampling area, and comparable irrigation and fertilization practices were applied across the areas. The annual chemical fertiliser inputs and the corresponding N, P_2_O_5_ and K_2_O inputs for the three cropping systems are presented in S4 Table (S1 File). Although nutrient inputs differed among the three crop categories, all three were distributed across the soil-texture sampling areas and were subjected to comparable agricultural management. Therefore, crop type, irrigation, and fertilization management were regarded as common background factors rather than independent experimental treatments in this study.For each soil texture, three sites were chosen, and three replicate test plots (4 × 9 m) were established at each site, totalling 36 test plots. These were categorised into Sand (n = 9), Clay Loam (n = 9), Clay (n = 9), and Loam (n = 9). Sampling was conducted in January and March 2024 (the dry season was chosen to provide a stable baseline for comparing texture- and depth-related nutrient stoichiometry without the confounding influence of storm-driven runoff and leaching events that dominate the wet season) using a five-point sampling technique. A soil auger was employed to obtain samples from three soil strata (0–20 cm, 20–40 cm, and 40–60 cm) in each plot. Samples were taken from the centre and each of the four corners of each plot, which were combined into a bulk sample weighing approximately 2 kg for the measurement of physicochemical soil parameters. In total, 216 soil samples were obtained. All soil samples were promptly passed through a 2-mm mesh to eliminate stones, root fragments, and large organic residues, ensuring thorough homogenisation of the samples. The samples were subsequently separated into three aliquots. One aliquot was transferred to an icebox and delivered to the laboratory for storage at 4°C, pending enzymatic analysis [32], soil moisture content, DOC, and AN. The second subsample was air-dried under ambient conditions and subsequently utilised for the analysis of soil texture, pH, and AP levels. The remaining subsample was pulverised and passed through a 0.149 mm sieve to analyse SOC, TP and TN contents.

**Fig 1:**
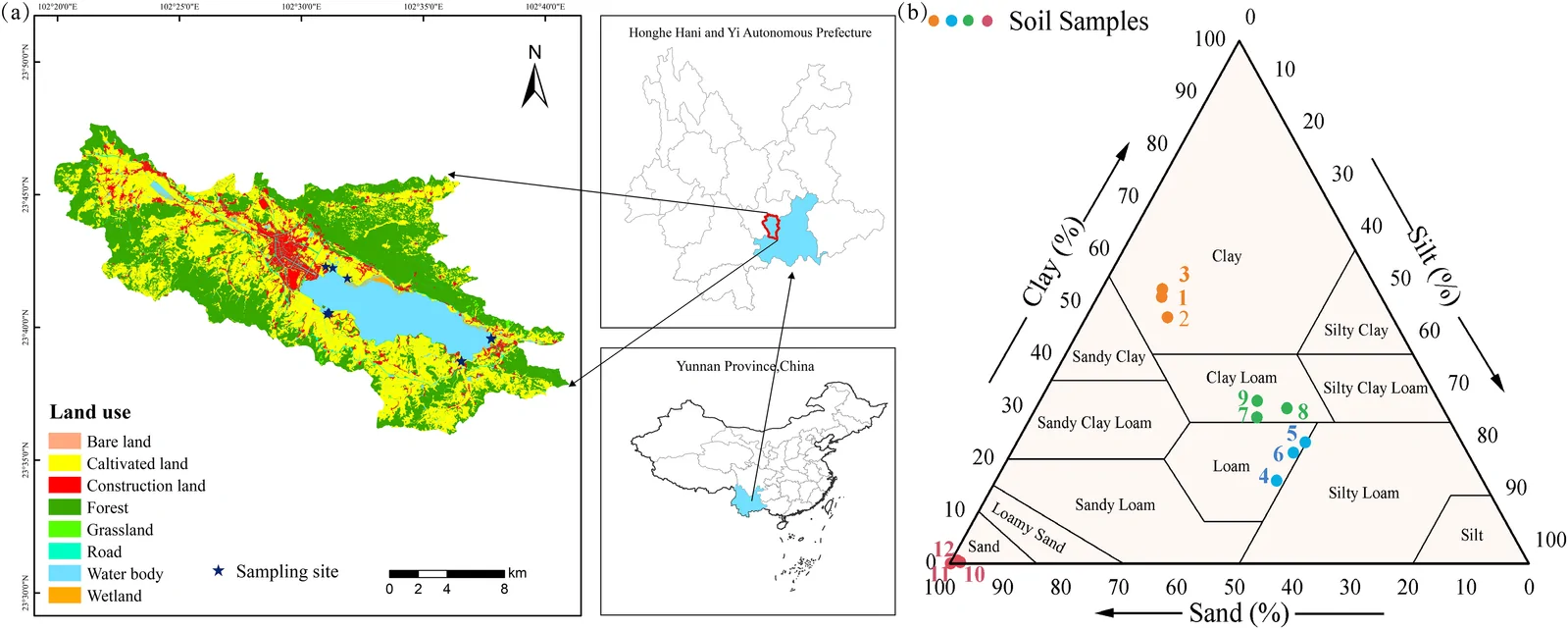
Map showing the location of the study area and soil texture classification. (a) Geographical location of the study area and distribution of sampling locations in Clay, Loam, Clay Loam and Sand areas (n = 12 × 3, there are 3 points in each of the 4 directions in the map, for a total of 12 points, with each point containing 3 test areas); (b) Classification of soil texture in the test sites. Note: The map of China is generate dusing the standard map data from the Standard Map Service of the Ministry of Natural Resources (Approval No.: GS (2019) 3333).

### 2.3 Soil physical and chemical properties

Soil particle composition (clay, sand, silt) was determined using a laser particle size analyser (SALD-2300, Shimadzu, Japan). Texture classes were assigned with the USDA soil-texture triangle using clay (<0.002 mm), silt (0.002–0.05 mm), and sand (0.05–2 mm) fractions. The soil moisture content was determined by the oven-drying method (drying at 105°C for 24 hours) and calculated as the mass difference between wet and dry soil [32]. Soil pH was measured in a 1:2.5 (w/v) soil-to-water suspension with a pH metre (RayKol AT300, China). SOC and total carbon (TC) contents were measured with a carbon/nitrogen analyser (multi N/C 3100, Analytik Jena, Germany). Within 48 hours of sampling, soil AN was extracted using a 1 mol·L^–1^ KCl (aq) solution at a 1:5 (w/v) soil-to-liquid ratio. The contents of nitrate N (NO_3_^–^-N) and ammonium N (NH_4_^+^-N) in the filtrate were determined with a fully automated intermittent chemical analyser (Clever Chem Anna, Germany). DOC was extracted with deionised water at a 1:5 (w/v) soil-to-water ratio, filtered through a 0.45 μm membrane, and measured with a TOC/TN analyser (multi N/C 3100, Analytik Jena). For TP and TN analyses, soil samples were digested with H_2_SO_4_–H_2_O_2_; the resulting solutions were colourimetrically measured using a fully automated intermittent chemical analyser. The stoichiometric ratios of C, P and N in the soil were expressed as mass ratios and calculated based on the contents of SOC, TN and TP, including SOC:TN, TN:TP and SOC:TP. Additionally, the ratios of labile nutrient fractions (DOC:AP, DOC:AN and AN:AP) were calculated using the same method. For soil ecoenzymatic activity analysis, we used a fluorometric microplate enzyme assay [33] to quantify the activities of the following five hydrolases valuable for the cycling of C, N, and P: β-1,4-glucosidase (BG), cellobiohydrolase (CBH), β-1,4-N-acetylglucosaminidase (NAG), leucine aminopeptidase (LAP), and alkaline phosphatase (ALP). Subsequently, 91 mL of 50 mM sodium acetate buffer (pH 7.8) and 2.75 g of fresh soil were mixed to create a suspension, and 200 μL of each suspension was pipetted into each well of a 96-well plate while being continuously homogenised with a magnetic stirrer. This procedure has been comprehensively described by Chen et al. [33]. The enzyme activities (nmol·h^−1^·g^−1^) were calculated as nmol substrate converted per mL of sample. Microbial metabolic limitation was quantified by calculating the vector length and vector angle of extracellular enzyme activities following Moorhead et al. [34]. The coordinates x and y were calculated using Eqs (1) and (2), respectively. Vector length (Eq (3)) indicates relative microbial C limitation, which increases with vector length. Vector angle (Eq (4)) indicates relative microbial N/P limitation: angles > 45° indicate P limitation, whereas angles < 45° indicate N limitation. Increasing vector angle indicates stronger relative P limitation and weaker relative N limitation [34]:


$$\begin{array}{c} x=ln(BG+CBH)/ln(NAG+LAP) \end{array}$$ (1)

$$\begin{array}{c} y=ln(BG+CBH)/lnALP \end{array}$$ (2)

$$\begin{array}{c} \text{Relative\textasciitilde{}C\textasciitilde{}limitation}=\text{\textasciitilde{}Length}=\sqrt{\left(x^{2}+y^{2}\right)} \end{array}$$ (3)

$$\begin{array}{c} \text{N/P\textasciitilde{}limitation\textasciitilde{}}=\text{\textasciitilde{}Angle}\left(o\right)={\text{atan}}^{2}\left(x,y\right)\times 180/\pi \end{array}$$ (4)

### 2.4 Statistical analyses

Two-way analysis of variance (ANOVA) was employed to investigate the primary and interactive effects of soil depth and texture on the contents of SOC, TP, TN, DOC, AP, and AN, as well as their stoichiometric ratios. Before conducting two-way ANOVA, the Shapiro-Wilk test was conducted to assess the data’s normality. To find variations in soil textures and layers, Tukey’s post hoc test was employed. SPSS 21 (IBM, Armonk, NY, USA) was employed to conduct all analyses. Mantel test analysis (was used as a distance-based preliminary screening tool to visualise the overall association structure between soil properties, depth and texture, and was intentionally supplemented by the standard pairwise correlation results presented in the same figure) was performed using R (version 4.4.2). SPSS AMOS 26 (Amos Development, Crawfordville, FL, USA) was employed to conduct path analysis to evaluate the direct and indirect relationships between soil texture, depth, elemental content, and specific stoichiometric ratios. The model was fitted to the measured data using maximum likelihood estimation. The goodness-of-fit index, p-values, χ^2^-values, and root mean square error of approximation were used to evaluate the model’s fit. A significance criterion of p < 0.05 was used for all tests. For path analysis, soil texture was represented by clay content (%) as a continuous proxy, following standard pedometric practice. Path analysis rather than SEM was chosen because the model contains only observed variables with no latent constructs. Prior to analysis, pairwise correlations among predictors were checked to avoid multicollinearity (S3 Table (S1 File), Fig 5). Because the data are observational, path coefficients represent significant associations under the model structure rather than confirmed causal effects.

**Fig 2:**
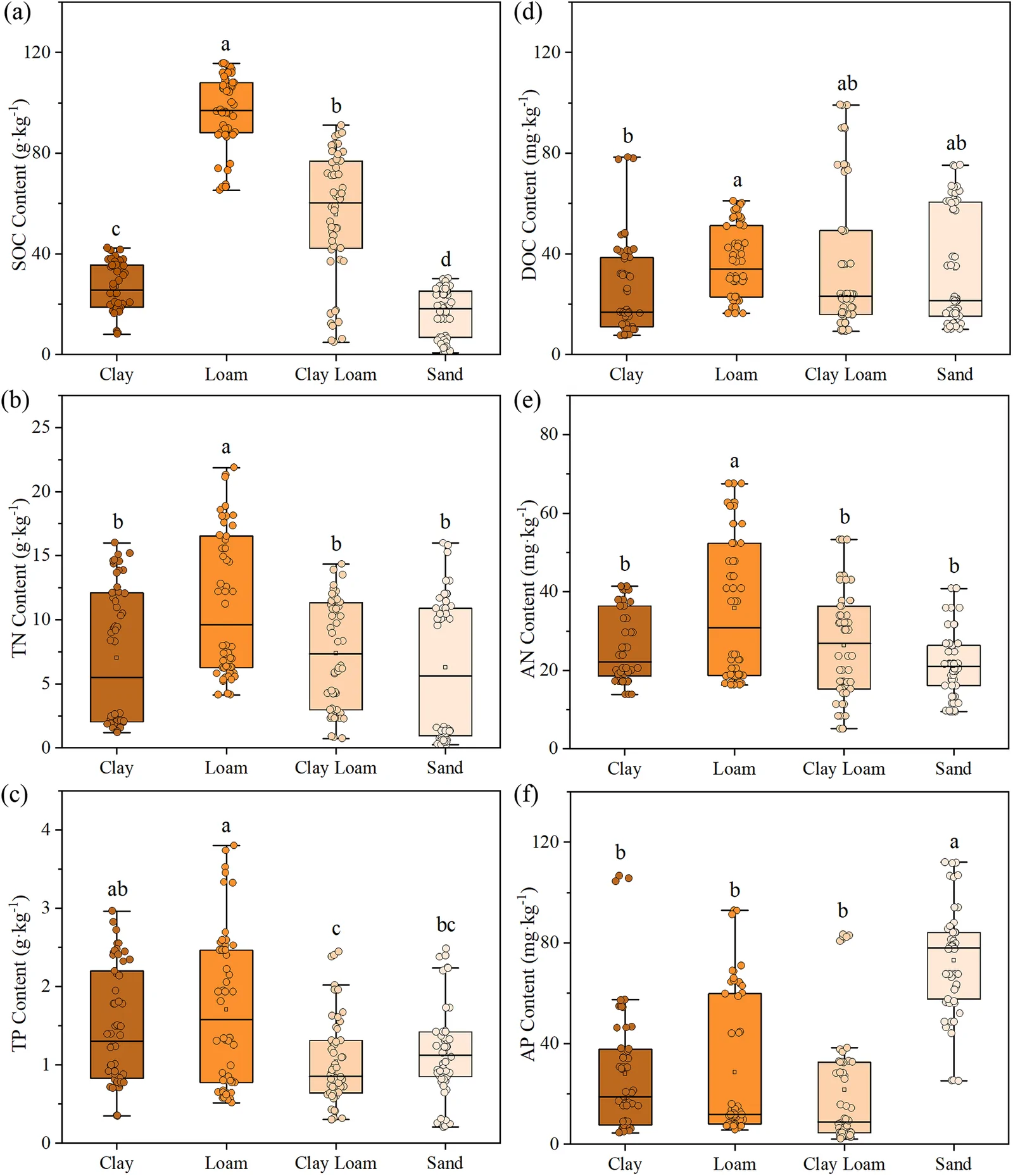
Distinct farmland soil textures in the low-latitude plateau lake basin exhibit variations in soil (a) SOC, (b) TN, (c) TP, (d) DOC, (e) AN and (f) AP contents. Error bars represent means ± standard errors. Diverse letters signify substantial disparities among various soil textures (p < 0.05, ANOVA).

**Fig 3:**
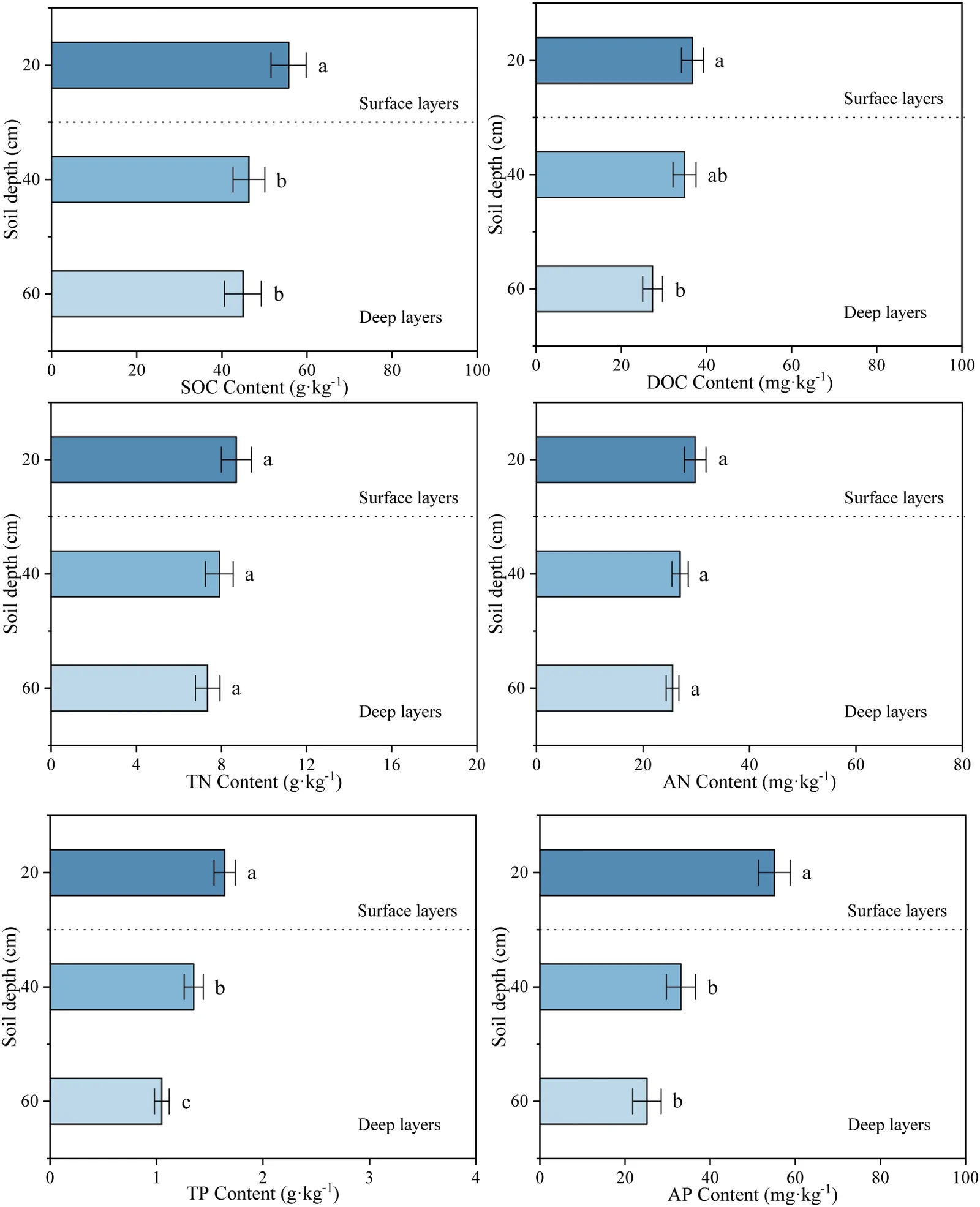
Available and total C, P and N vertical distributions in farming soils in the low-latitude plateau lake basin. Diverse letters signify substantial disparities among various soil layers (p < 0.05, ANOVA). Error bars represent means ± standard errors.

**Fig 4:**
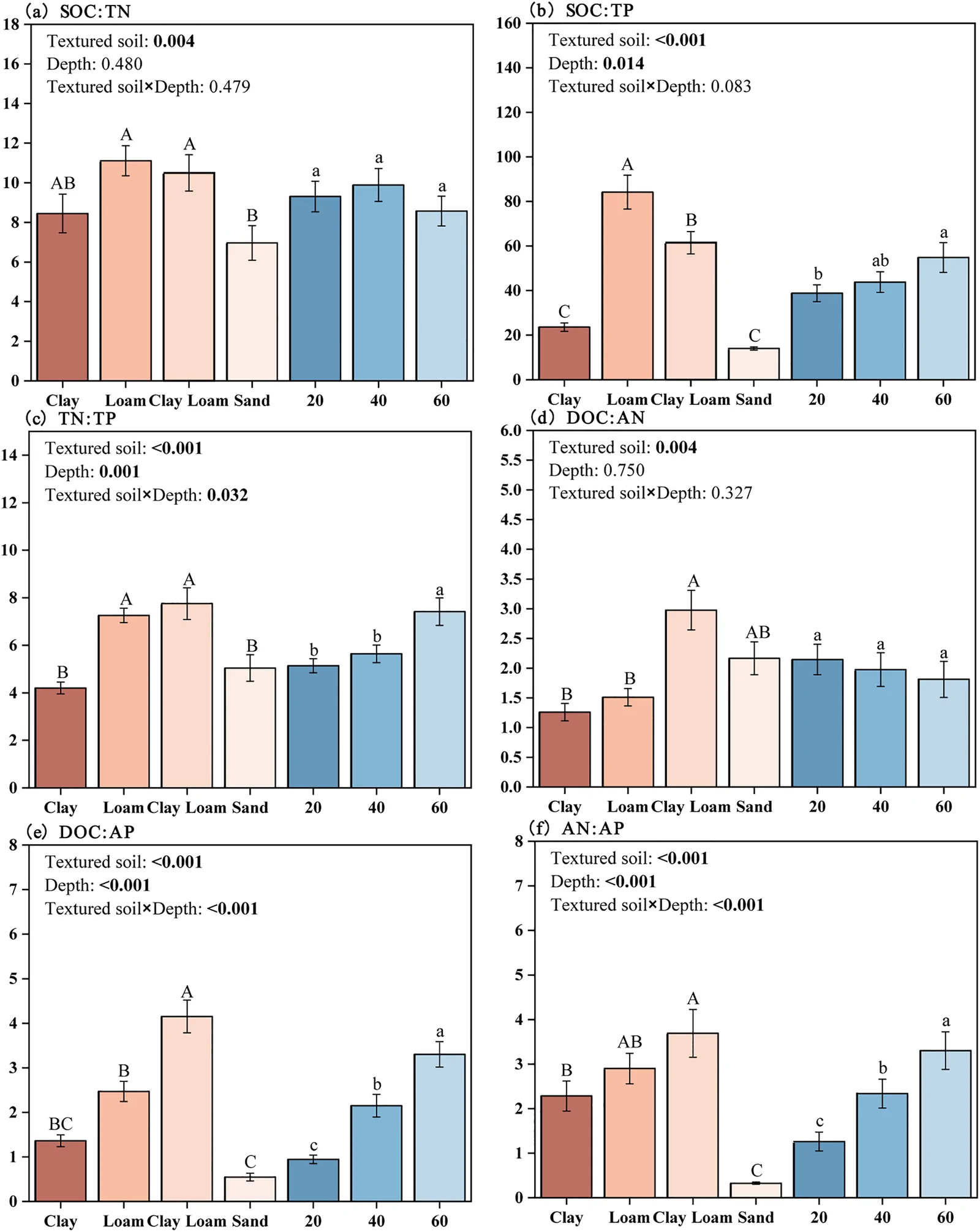
The stoichiometric ratios of available and total C, P and N vary with soil depth and texture. Variations in (a) SOC:TN, (b) SOC:TP, (c) TN:TP, (d) DOC:AN, (e) DOC:AP and (f) AN:AP across three layers and among four textures. The columns denote means ± standard errors. The abscissa denotes soil textures and soil layers, with pink and blue columns representing different soil textures (Clay, Loam, Clay Loam, Sand) and layers (0–20 cm, 20–40, and 40–60 cm). Significant differences are indicated by distinct letters (p < 0.05; capital letters for varying soil textures and small letters for differing depths).

## 3 Results

### 3.1 Available and total C, P and N contents in soils of varying texture

The contents of AN, DOC, AP, SOC, TP and TN, across the different soil textures studied ranged from 21.54 to 35.82 mg·kg^–1^, 25.55 to 36.63 mg·kg^–1^, 21.65 to 72.96 mg·kg^–1^, 17.01 to 96.19 g·kg^–1^, 1.04 to 1.70 g·kg^–1^ and 6.27 to 11.26 g·kg^–1^, respectively (Fig 2). The TN and SOC contents decreased in the following order: Loam > Clay Loam > Clay > Sand. Regarding soil TP, Clay Loam exhibited the lowest levels. The DOC and AN contents were significantly higher in Loam than in other soil textures. Conversely, Sand showed a significantly higher AP content relative to other soils.

### 3.2 Vertical stratification of available and total C, P and N contents in soil profiles

The availability and total of C, P and N in the soil diminished with increasing depth. The levels of SOC, TP, DOC and AP were markedly elevated in the surface soil compared to the deeper soil (p < 0.05). However, no significant differences in TN and AN contents were observed across the various soil layers (Fig 3). The 0–20 cm and 40–60 cm layers had the highest and lowest values for SOC, TP, TN, DOC, AP and AN, respectively, with mean values of 55.68 and 44.97 g·kg^–1^, 8.70 and 7.35 g·kg^–1^, 1.64 and 1.05 g·kg^–1^, 36.70 and 27.34 mg·kg^–1^, 29.78 and 25.57 mg·kg^–1^, and 55.05 and 25.14 mg·kg^–1^. Additionally, soil pH increased with depth (S3 Table, S1 File). Soil moisture content differed markedly among soil textures and showed distinct vertical patterns (S1 Fig): Loam exhibited the highest moisture content, followed by Clay Loam and Clay, whereas Sand showed the lowest moisture content across all three soil layers. Within a given texture, soil moisture increased with depth in Loam and Clay Loam, whereas it remained relatively stable across depths in Clay and Sand.

### 3.3 Stoichiometric ratios of C, P and N in soil: Variations with depth and texture

Results from a two-way ANOVA analysis of variance showed that the SOC:TN, TN:TP and SOC:TP varied significantly between soil types with varying textures. (p < 0.01, Fig 4a–4c). The soil SOC:TP reached its maximum in Loam, whereas the SOC:TN was at its minimum in Sand (p < 0.01, Fig 4a–4b). There was a consistent pattern across soil textures for SOC:TP and SOC:TN: Loam > Clay Loam > Clay > Sand. There was a significant difference in SOC:TP and TN:TP among soil layers (p < 0.05, Fig 4b–4c). The 40–60 cm layer has the highest SOC:TP and TN:TP, followed by the 20–40 cm and 0–20 cm layers. In contrast, the SOC:TN did not significantly vary across soil layers. However, significant interactions between soil depth and texture were observed, affecting the TN:TP (p < 0.05, Fig 4c).

AN:AP and DOC:AP was significantly affected by soil depth and texture (p < 0.05, Fig 4e–4f). Notably, DOC:AN, AN:AP and DOC:TP exhibited significantly elevated levels in Clay Loam compared to Clay (p < 0.05, Fig 4d–4f). A consistent pattern of AN:AP and DOC:AP was observed across several soil textures: Clay Loam > Loam > Clay > Sand. The AN:AP and DOC:AP exhibited substantial differences throughout the soil profile (p < 0.001, Fig 4e–4f). The highest ratios were seen in the 40–60 cm soil layer, followed by the 20–40 cm and 0–20 cm layer. Conversely, DOC:AN exhibited no substantial variation among soil layers (Fig 4d).

Interestingly, the interaction between depth and texture was significant (p < 0.05) for available nutrient ratios (AN:AP, DOC:AP), whereas it was not evident for total nutrient ratios (SOC:TN, SOC:TP). This preliminary finding supports the hypothesis 2 that available nutrients exhibit greater sensitivity to changes in the physical environment (Fig 4).

### 3.4 Interrelationships between soil properties, stoichiometric ratios, soil texture and depth

Soil texture was found to have a strong positive correlation with SOC (p < 0.01) according to Mantel’s test analysis, and a substantial negative correlation with pH and AP. A positive and substantial correlation (p < 0.05) was also found between soil texture and TP, TN, AN, AN:AP, DOC:AP, SOC:TP and SOC:TN. The relationship between soil depth and pH, AN:AP, DOC:AP and TN:TP was positive (p < 0.05), while the relationship was negative with DOC, AP, TP and SOC. Soil texture was shown to have no relationship with DOC, DOC:AN or TN:TP (Fig 5). Finally, soil depth had less of an impact on SOC, TP and TN levels and stoichiometric ratios than soil texture.

Path analyses were conducted to examine the possible direct and indirect associations between soil depth and texture on the stoichiometric ratios of C, P and N. Soil texture showed indirect associations with SOC:TN, TN:TP, SOC:TP, AN:AP and DOC:AP, while it influenced DOC:AN both directly and indirectly. Depth exerts indirect effects on SOC:TN and DOC:AN, as well as direct and indirect impacts on TN:TP, SOC:TP, and DOC:AP, including TP, SOC:TP, AN:AP and DOC:AP. SOC:TP and SOC:TN is influenced by SOC via the effects of soil depth and texture on TP (Fig 6a–6b). DOC:AP and DOC:AN are affected by AN via the impacts of soil depth and texture on TP (Fig 6d–6e). Furthermore, TN:TP was influenced by soil texture mostly via SOC and AN (Fig 6c). Soil depth and texture affected the AN:AP via AN content (Fig 6f).

**Fig 5:**
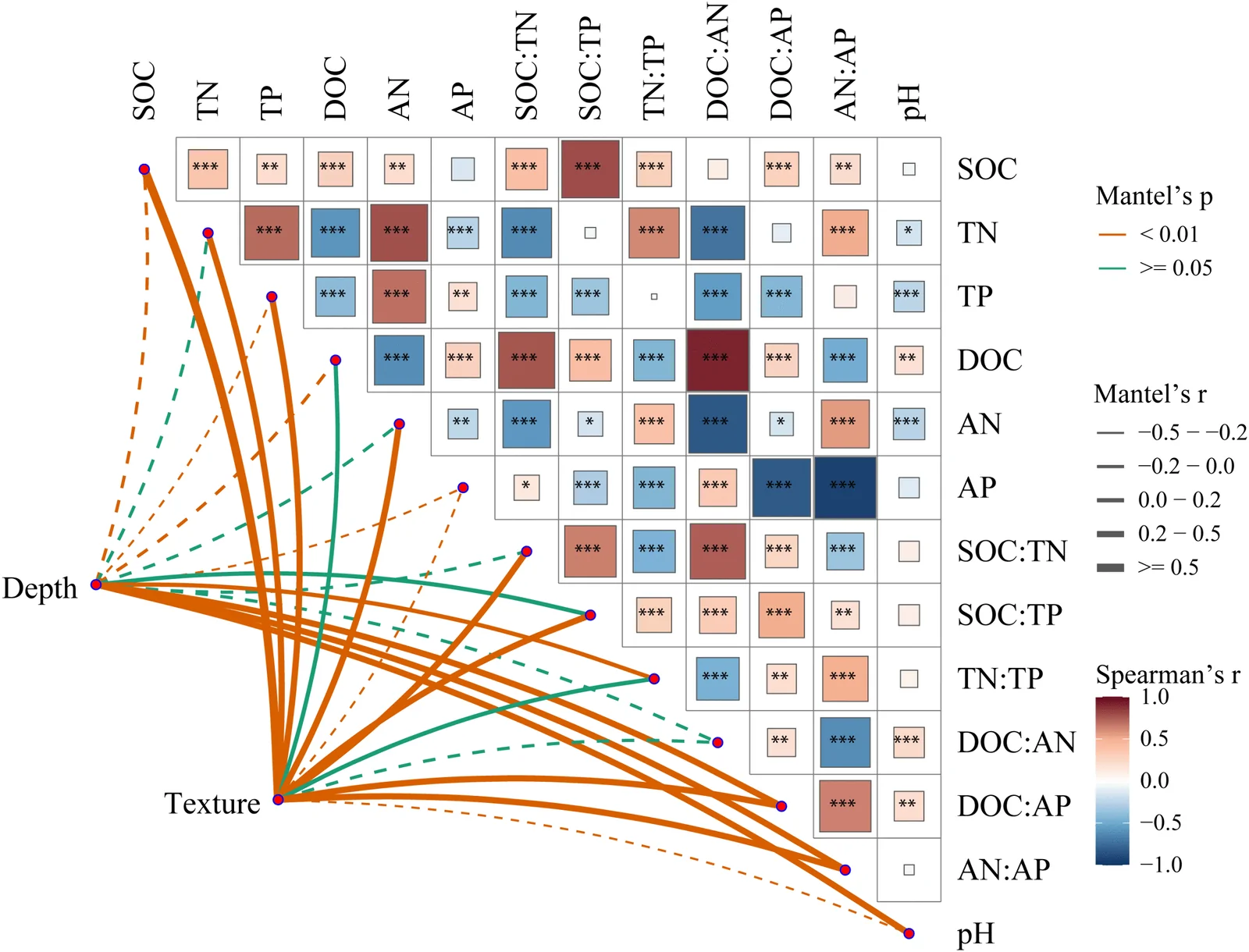
Correlation plot of soil properties and stoichiometric ratios of farmland soils of different depth and textures s in the valley of Yilong Lake, China. Red areas indicate a significant correlation, blue areas indicate no significant correlation; solid lines indicate a positive correlation, and dotted lines indicate a negative correlation. Depth, soil depth. Texture, soil texture type (soil texture was assigned a value based on clay particle content).

**Fig 6:**
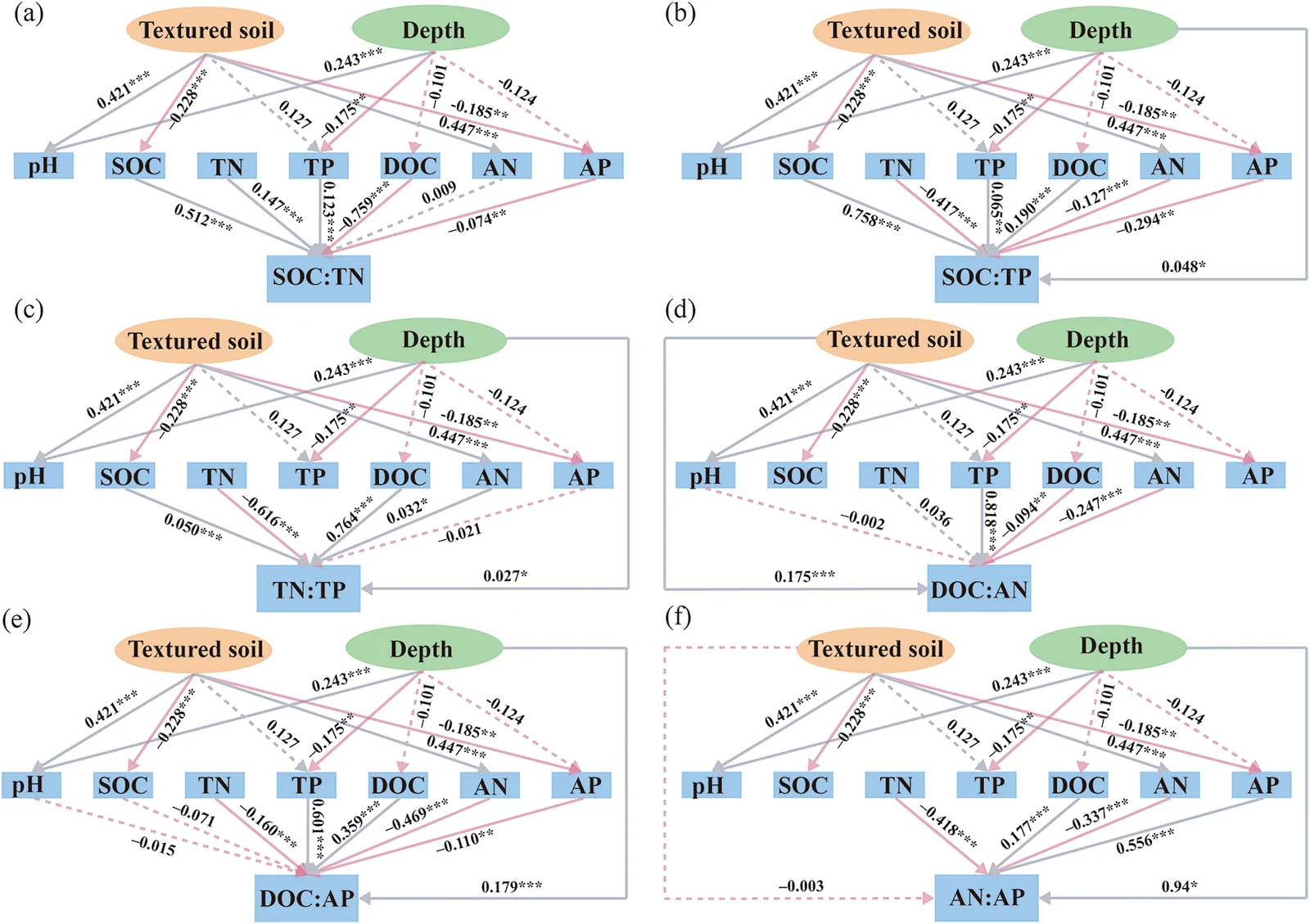
Path analysis of hypothesized associations among soil depth, texture, pH, C, P and N content, and stoichiometric ratios of available and total soil. The model fit parameters were as follows: for (a) SOC:TN, p = 0.25, χ^2^ = 28.16, RMSEA = 0.02, GFI = 0.94; (b) SOC:TP, p = 0.31, χ^2^ = 24.08, RMSEA = 0.02, GFI = 0.95; (c) TN:TP, p = 0.52, χ^2^ = 23.89, RMSEA = 0.03, GFI = 0.93; (d) DOC:AN, p = 0.51, χ^2^ = 23.96, RMSEA = 0.02, GFI = 0.94; (e) DOC:AP, p = 0.48, χ^2^ = 22.91, RMSEA = 0.01, GFI = 0.92; (f) AN:AP, p = 0.56, χ^2^ = 20.24, RMSEA = 0.01, GFI = 0.93. Soil texture was assigned a value based on clay particle content. The grey line represents a positive and the red line a negative. The solid lines show statistically significant effects (p < 0.05), the dashed lines show effects that are not statistically significant, and the symbols *, **, and *** denote p < 0.05, p < 0.01 and p < 0.001, respectively.

## 4 Discussion

### 4.1 Associations between available and total C, P and N in Southwestern China

Soil nutrient content and its stoichiometric ratios serve as critical indicators for assessing soil organic matter composition and quality, which are important for understanding ecosystem health and sustainability. This study revealed that slightly alkaline soils (S3 Table, S1 File) facilitate the formation of Fe/Al oxides, significantly influencing soil nutrient dynamics. Among different soil textures, Loam showed a notably higher contents in C, P and N storage. It exhibited the highest content of SOC (96.19 g·kg^–1^) versus TN (11.26 g·kg^–1^), along with a significantly elevated SOC:TP (84.17) compared to other textures. As shown in S1 Fig, the high moisture content of Loam soil promotes the conversion of readily decomposable C into stable SOC by microorganisms, thereby increasing the C:N and C:P. In the same time, it indicates that Loam has a high capacity for C and N sequestration but may face potential P limitation (S2 Fig), highlighting the potential need for sustainable P management strategies to maintain soil fertility and ecosystem balance.

Compared with Loam, Clay Loam had significantly lower content of both TP (1.04 versus 1.70 g·kg^–1^) and AP (21.65 versus 28.56 m g·kg^–1^), resulting in a higher DOC:AP (4.15 versus 2.47). These patterns indicate restricted P availability, potentially associated with phosphate sorption by Fe/Al oxyhydroxides under clay-rich and weakly alkaline conditions (pH 7.81) [35,36]. However, low P availability does not necessarily mean that P is the predominant constraint on microbial metabolism.

The divergence in microbial nutrient limitation between these two textures arises from the interplay between structural and non-structural factors. Structurally, the higher clay content in Clay Loam enhances physicochemical stabilization of organic matter through clay sorption and micropore protection, thereby retarding nitrogen mineralization and restricting the supply of bioavailable nitrogen [14,15]. Non-structurally, the weakly alkaline pH (7.81) not only promotes PO_4_^3–^ fixation by Fe/Al oxyhydroxides but also favors nitrification, which, under moist conditions, increases the risk of nitrogen loss via leaching or ammonia volatilization [35]. Consequently, despite lower absolute P content, Clay Loam exhibited a substantially higher DOC:AN (2.98 versus 1.51), reflecting a stoichiometric imbalance between DOC and AN. Drawing upon ecoenzymatic stoichiometry theory [9,34], this C:N imbalance signals that microbes face a relative nitrogen deficit when decomposing organic matter, triggering preferential allocation to N-acquiring enzymes. This metabolic shift was corroborated by the extracellular-enzyme vector angle of 38.5° (< 45°), indicating relative microbial N limitation, whereas Loam showed an angle of 48.2° (> 45°), indicating relative microbial P limitation (S2 Fig).

Therefore, Clay Loam exhibited coexisting constraints on P availability and predominant relative microbial N limitation. The asynchronous changes in soil C, P and N dynamics illustrate that the nutrient functionally limiting microbial metabolism cannot be inferred from absolute nutrient stocks alone; indeed, TP may vary independently of SOC and TN [36]. Collectively, the convergence of stoichiometric and enzymatic evidence provides a robust, mechanism-oriented explanation for the observed N limitation in Clay Loam, underscoring the importance of considering resource stoichiometry when assessing microbial nutrient constraints across different soil textures.

Sand was characterized by significantly enriched AP content (72.96 mg·kg^–1^), but their SOC:TN (6.96) was the lowest among the four soil types. This low SOC:TN reflects a high risk of concurrent C and N loss, mainly due to the degradation of organic matter exacerbated by water and wind erosion in these loose-textured soils. Such a situation not only threatens soil quality but also impacts the C sequestration capacity and greenhouse gas emissions of the ecosystem. Therefore, implementing soil conservation measures, such as cover cropping and terracing, is valuable to mitigate C and N losses and enhance the sustainability of Sand soil-based agro-ecosystems.

Based on the nutrient categorization criteria of the Second China Soil Census (S2 Table, S1 File), the TN (> 2 g·kg^–1^) and TP (> 1 g·kg^–1^) contents of the four soil textures in the watershed exceeded the Class 1 standard, indicating relatively high total nutrient levels. However, except for Loam, which fell within nutrient Class 4, the AN content of other soil textures was below the Class 5 standard (< 30 mg·kg^–1^). The relatively high TN content in Loam, combined with the slightly alkaline soil environment that suppresses AN volatilization, contributed to its elevated TN accumulation. Regarding phosphorus, while the overall TP content met the standard threshold (> 1 g·kg^–1^), AP levels varied significantly across soil types. Sand, due to limited physical leaching, achieved the Tier 1 nutrient standard (>40 mg·kg^–1^) for AP, while the remaining soils met the Tier 2 standard (20–40 mg·kg^–1^), with Clay Loam showing the most pronounced AP depletion due to chemical fixation. These findings emphasize that soil texture plays a important role in shaping the nutrient distribution pattern in this region, and targeted nutrient management practices are necessary to optimize soil fertility and ensure sustainable agricultural production.

The study also observed a “surface aggregation” effect, where the surface layer (0–20 cm) had 23.80%, 56.67%, 34.26%, and 119.13% higher levels of DOC, AP, SOC and TP, respectively, compared to the deep layer (40–60 cm). This phenomenon can be attributed to the accumulation of plant root inputs and animal leftovers in the upper soil layer, where enhanced microbial activity promotes the buildup of SOC and DOC [21,23,37]. Unlike carbon, which is incorporated into the soil through biological fixation processes such as photosynthesis, phosphorus enters the soil via the weathering of primary minerals containing phosphorus [38,39]. In frequently cultivated agricultural soils, the intensity of disturbance and weathering decreases with depth, leading to a decline in TP content as soil depth increases [40]. Additionally, the formation of apatite from calcium carbonate in the slightly alkaline soil environment increases P retention in the soil as pH rises, reducing P utilisation efficiency. These findings (Fig 2, S3 Table, S1 File) highlight the importance of considering soil depth when evaluating soil nutrient availability and ecosystem health.

Notably, contrary to previous research, this study found no significant variation in TN between soil strata [21,23]. This discrepancy may be attributed to the mildly alkaline soil characteristics and the sampling period during winter and spring, when the local climate is warm and extremely arid. Under these conditions, the use of ammonia-based fertilisers, such as urea, likely intensifies ammonia volatilization from the soil surface, reducing nitrogen levels in the upper soil layer and thus minimizing the difference in TN across soil layers. This result underscores the complex interactions between soil properties, climate, and human activities in influencing soil nutrient dynamics, which should be considered in sustainable nutrient management strategies.

Regarding the relationships between soil nutrients, positive correlations were observed between soil DOC and SOC, AN and TN, as well as AP and TP (Fig 5). Elevated SOC and TN levels enhance the availability of DOC and nitrogen sources in the soil, promoting the transformation and accumulation of soil C and N, and consequently increasing DOC content and generating AN. This indicates that managing nitrogen nutrients should take into account SOC content to optimize nitrogen use efficiency and reduce environmental impacts. Understanding these complex relationships is valuable to developing sustainable nutrient management practices that balance soil fertility, plant nutrition and environmental protection.

### 4.2 Impact of diverse soil depths and textures on the stoichiometry of total C, P and N in subtropical soils of Southwestern China

This study delved into the influence of soil texture on the stoichiometric ratios of C, P and N across various soil profile gradients, with a specific focus on low-latitude plateau agroecosystems. The research findings support our proposed Hypothesis 1, emphasizing the significant role of soil texture in shaping soil physicochemical properties and stoichiometric relationships, which are valuable for understanding ecosystem health and environmental sustainability.

Among different soil textures, nutrient-rich Loam demonstrated notably higher SOC:TN and SOC:TP, along with elevated TN and SOC contents, compared to Sand (Figs 2 and 4). This difference is likely due to the fine-grained nature of Loam—fine-grained soils typically contain more nutrients than coarse-grained soils (S1 Table, S1 File). Fine-grained soils generally possess a greater capacity for nutrient retention due to their enhanced surface area, allowing for more effective adsorption and aggregation of ions and organic matter [20]. Moreover, the superior water-holding capacity of Loam ensures a more consistent water supply for plants, which in turn stimulates plant primary productivity and increases soil nutrient inputs. These factors collectively contribute to the higher nutrient content and distinct stoichiometric ratios observed in Loam. Across various soil textures, the trends in SOC:TN and SOC:TP closely mirrored those of soil moisture content (S1 Fig), further emphasizing the interconnections between soil physical properties and nutrient dynamics.

The SOC:TN across different soil textures in this study ranged from 6.96 to 11.11, values below or around the average for Chinese soils (11.9) as reported by Tian et al. (2010). The C:N of soil organic matter, which is inversely related to the decomposition rate, was comparable to the standard C:N (8.67) found in Chinese agricultural soils [41]. Soils with lower C:N tend to mineralize more rapidly, likely a consequence of prolonged anthropogenic activities in agricultural lands. In the Yilong Lake basin, practices such as leaving crop residues in the field and applying bio-organic fertilisers may have enhanced soil porosity, increased microbial activity, and accelerated the decomposition of organic matter. This process not only reduces the formation of humus but also releases more nitrogen, which aligns with the observation that the nitrogen content of farmland soils in this basin exceeded the Level 1 nutrient standard. From an environmental sustainability perspective, these management practices have both positive and negative implications. While they contribute to soil fertility in the short term, the increased nitrogen release may also pose a risk of nitrate leaching, as indicated by the stoichiometric principle that soils with a C:N below 30 are more prone to nitrate loss. Our results (Fig 4a–4c) indeed suggest a potential threat of severe nitrogen leaching in the agro-ecosystem soils of this watershed, highlighting the potential need for more sustainable nitrogen management strategies to mitigate environmental impacts.

The soil TN:TP in this study (4.20–7.75) (Fig 4c) were close to the Chinese average (5.2), yet a distinct pattern emerged with depth, where the TN:TP in the deep soil layer (7.41) was significantly higher than that in the surface layer (5.14), indicating that vertical differentiation of phosphorus is more pronounced (with deep-layer TP significantly reduced due to fixation and leaching), while nitrogen mineralisation and fixation remain relatively stable (Fig 3). Additionally, Clay Loam and Loam exhibited notably higher TN:TP compared to other soil textures. Path analysis revealed that soil texture influenced TN:TP primarily through its effect on SOC content, while soil depth had a direct impact on this ratio. This finding contrasts with previous research in the Poyang Lake Plain [26] and the Songhua River Plain [42], where the TN:TP was predominantly regulated by TP. The discrepancy can be attributed to the unique characteristics of lakeshore farmland soils in the low-latitude plateau basin, which have higher TC and SOC contents compared to plain soils. The elevated SOC content affects the SOC:TN, thereby influencing nitrogen release dynamics. This highlights the complexity of nutrient cycling in different ecosystems and emphasizes the importance of considering site-specific factors when formulating strategies for managing agricultural non-point source pollution. In the context of the low-latitude plateau, a holistic approach that integrates the regulation of C, P and N is may be required to optimize stoichiometric ratios and minimize nutrient losses, thereby protecting water quality and enhancing ecosystem sustainability.

The SOC:TP, along with SOC and TN levels, was significantly higher in Loam compared to the other three soil textures (Figs 2a–2b and 4b). The finer texture of Loam enables greater retention of soil organic matter, as it facilitates stronger associations between organic materials and minerals such as clays and iron or aluminum oxides [43]. This enhanced retention reduces the transport and decomposition of soil organic matter, contributing to the elevated SOC:TP. The wide range of SOC:TP values (14.03–84.17) (Fig 4b) observed in this study is consistent with previous research on Chinese soils [44]. The C:P serves as a valuable indicator indicator of soil phosphorus mineralization potential. Loam exhibited a much higher SOC:TP compared to other soil textures, suggesting a lower capacity for phosphorus mineralization. Typically, a low C:P promotes the release of phosphorus by microorganisms during organic matter decomposition, increasing soil available phosphorus. Conversely, a high C:P restricts microbial phosphorus release, leading to competition between microorganisms and plants for inorganic phosphorus, which can potentially limit plant growth. The SOC:TP of Loam in this study (84.17) exceeded both the Chinese (52.7) [44] and global (72.0) [8] terrestrial averages. This high ratio, primarily driven by the significantly higher SOC content in local Loam, indicates reduced net phosphorus mineralization rates. Phosphorus limitation may thus impede the microbial decomposition of organic matter in Loam, further highlighting the potential need for targeted phosphorus management strategies to improve soil fertility and support sustainable agricultural production.

Based on all soil samples collected from the 0–60 cm depth range, the average C:N:P in farmland soils of the Yilong Lake region was determined to be 36:6:1 (S3 Table, S1 File). The strong correlations among C, P and N contents (Fig 5) suggest that these three elements are coupled through shared biogeochemical processes. These processes include soil organic matter input, decomposition, and mineralization. This coupling implies that management practices altering one element may affect the cycling of the others. Therefore, an integrated nutrient management approach may be more appropriate than single-nutrient strategies.

The regional soil C:N:P of 36:6:1 is lower than the Chinese average for terrestrial soils (60:5:1) [44] and the global average (186:13:1) [8].Agricultural soils often deviate from natural stoichiometric equilibria because they receive substantial external nutrient inputs. The low C:P and N:P components of our ratio indicate relative P enrichment in the Yilong Lake basin. Alternatively, rapid C turnover may contribute to this pattern. Both mechanisms are consistent with our C:N and C:P results. Intensive agricultural management—including residue return, bio-organic fertiliser application, and P-containing compound fertilisers—has likely increased soil P availability and accelerated organic matter decomposition. These findings support Hypothesis 1. Soil texture and depth appear to influence the stoichiometric structure of this agroecosystem. They do so primarily by mediating SOC accumulation and its subsequent effects on N and P cycling, rather than by directly constraining nutrient pools.

### 4.3 Impact of diverse soil depths and textures on the stoichiometry of available C, P and N in subtropical soils of Southwestern China

N and P availability often pose significant constraints to agricultural productivity in subtropical regions [45]. While soil AN and AP are fundamental to crop growth, existing research on soil C:N:P stoichiometry has predominantly concentrated on total elemental contents. Empirical investigations into the dynamics of DOC and labile nutrient fractions within cultivated ecosystems remain notably limited. This study supported Hypothesis 2 by elucidating the complex relationship between soil texture, depth, and the stoichiometry of available nutrients, thereby providing valuable insights into ecosystem health and environmental sustainability.

Analysis of soil samples revealed distinct patterns in DOC partitioning across different soil textures. Loam exhibited significantly higher DOC contents compared to Clay, a result that suggests that the excessive presence of clay particles limits the bioavailability of soluble organic substrates that are may be required for microbial metabolism. In Clay, the fine particle structure and high cation exchange capacity can sequester organic compounds, reducing their accessibility to microorganisms. To enhance soil fertility for agricultural production in Clay of the study area, a combination of deep tillage and biochar application is recommended to improve soil permeability. Additionally, microbial inoculation can stimulate soil biological activity, facilitating nutrient cycling and plant uptake.

Clay Loam demonstrated elevated DOC:AP and DOC:AN relative to Clay and Loam (Fig 4d), indicating enhanced bioavailability of these nutrients. The high pH characteristic of Clay Loam creates an optimal edaphic environment for crops such as cauliflower, lettuce, asparagus, sugar beets, and soybeans, which exhibit a preference for slightly alkaline conditions. Implementing moderate reductions in fertiliser and pesticide applications can further promote sustainable cultivation practices, minimizing environmental impacts while maintaining crop productivity. Across all soil textures, the DOC:AP and AN:AP followed a consistent hierarchical order: Clay Loam > Loam > Clay > Sand (Fig 4e–4f). Although SOC:TP and SOC:TN also varied among soil textures, their patterns did not fully align with those of the available nutrient ratios, highlighting the complex interplay between total and available nutrient pools.

Path analysis revealed that DOC:AN was indirectly modulated by AN content and directly influenced by soil texture (Fig 6d). The elevated AN levels in Loam were a primary driver of this variation (Fig 2). In contrast, coarse-textured sandy soils exhibited higher rates of nitrate leaching compared to Clay Loam, attributable to enhanced water infiltration rates and reduced denitrification potential [17]. These hydrological and biochemical factors collectively contributed to significant disparities in AN content among soil textures. The regulation of AN:AP and DOC:AP by soil texture occurred primarily through AN and AP availability. Sand displayed significantly lower AN:AP and DOC:AP, primarily due to the dilutive effect of their high AP content (Fig 2). This finding underscores the potential need for targeted AN supplementation in sandy soils of the watershed. Accordingly, Sand farmlands in the Yilong Lake basin, characterized by high AP but low AN levels, are well-suited for cultivating crops with relatively high P and low N requirements, such as onions and leeks.

With increasing soil depth, the AN:AP and DOC:AP exhibited an upward trend, paralleling the patterns observed for TN:TP and SOC:TP. Conversely, the DOC:AN decreased with depth, primarily regulated by AN availability (Fig 4d). Deeper soil layers showed reduced AN and DOC contents, as well as lower DOC:AN (Figs 2d–2f and 4d), likely resulting from ammonia volatilization and nitrate leaching in the surface layer, exacerbated by tillage and irrigation practices. In contrast, the surface soil layer had lower AN:AP and DOC:AP compared to deeper layers, primarily influenced by TP and AP dynamics (Fig 6e–6f). The accumulation of TP and AP in the surface layer, a consequence of anthropogenic fertilization practices in agroecosystems, contrasts with findings from forest and grassland ecosystems, where phosphorus distribution is more homogeneous with depth [45–48]. These findings support the third hypothesis proposed in this study: texture primarily influences the stoichiometric ratio of available nutrients indirectly through SOC and AN. They simultaneously underscore the considerable influence of soil depth and texture on available nutrient distribution, with cascading effects on plant nutrient acquisition and soil nutrient loss via leaching.

Based on the above findings, this paper proposes a nutrient management framework tailored to different soil textures, with a view to enhancing the sustainability of agricultural systems in low-latitude plateau lake catchment areas.

For Loam, maintaining a balanced input of C, P and N is may be necessary to prevent stoichiometric imbalances. Regulating P application rates and promoting C sequestration can enhance soil fertility while reducing the risk of nutrient loss. In deeper soil layers, the use of slow-release fertilisers and straw incorporation strategies may help to reduce nutrient leaching and improve P utilisation efficiency. Establishing vegetative buffer zones along field margins, implementing cover cropping systems, and adopting precision irrigation techniques can further reduce surface runoff and associated nutrient losses.

In Clay Loam areas, applying humic acid-based amendments can modify soil structure and reduce the fixation of phosphorus by iron oxides, thereby enhancing P availability. The adoption of slow-release and controlled-release fertilisers may help to reduce N and P losses during intense rainfall events, while increased application of biochar-based organic fertilisers can elevate soil organic matter content and facilitate nutrient cycling.

For Clay, a combination of deep tillage and biochar application can improve soil aeration and water infiltration. Microbial inoculation and the incorporation of crop residues, along with the cultivation of green manure crops through intercropping systems (such as integrating zoysia or clover with vegetable crops), can enhance soil biological activity and nutrient retention. Prior to the rainy season, constructing stepped vegetation belts can increase the hydraulic residence time of runoff, reducing flow velocity and promoting the sedimentation of particulate matter and pollutant adsorption.

In Sand, amending with well-decomposed organic fertilisers (such as cow dung and straw compost) can increase soil organic matter content. Adding biochar at a rate of 5–10% can improve soil structure and water-holding capacity, while well-timed mulch tillage and integrated water and fertiliser techniques may help to reduce C and N losses. The deep application of urease inhibitors can further reduce N volatilization and mitigate wind erosion-induced nutrient losses.

### 4.4 Limitations and future research

These patterns are valuable for understanding soil nutrient utilisation and the risk of nutrient export in agricultural fields with varying soil textures near lakes on the low-latitude plateau. Furthermore, the findings provide a foundation for modelling elemental cycling in agricultural fields near lakes on regional and global scales. However, future research should address three key uncertainties to advance sustainable resource use for food, water, and climate security: (1) Nutrient loss mechanisms: Although the effects of soil depth and texture on nutrient content have been examined, the single-season study design limits inferences regarding variability during the rainy season, leaving the pathways of loss (such as runoff and leaching) and their relationship with soil properties unclear. Integrating hydrological modelling and nutrient loss monitoring will clarify these mechanisms, providing technical support for mitigating agricultural non-point source pollution; (2) Microbial community impacts: Soil microbes are central to nutrient cycling, yet this study did not examine microbial community structure or its role in nitrogen transformation. Human-induced nutrient loss reduces microbial biomass/diversity, altering nitrogen mineralization and retention [49,50]. Future work should combine microbiological analyses to elucidate microbial contributions to soil nutrient dynamics and cycling; (3) Smallholder farm management: Develop simple, accessible practices by integrating research insights into precision fertilization techniques. This will foster environmentally friendly strategies to reduce agrochemical inputs, enhance productivity, and build sustainable farmland ecosystems, aligning with food, water, and climate security goals.

## 5 Conclusion

This study identifies soil texture and depth as key physical drivers regulating nutrient cycling in highland lakeshore agro-ecosystems. Total nutrients are primarily governed by texture, whilst available nutrient ratios are significantly influenced by the interaction between texture and depth. Path analysis revealed that texture was indirectly associated with available nutrient balance through SOC and AN pools, whereas depth directly alters nutrient ratios by affecting TP. This finding highlights the potential value of zoning, stratification, and precision management based on soil physical properties, providing valuable scientific evidence for mitigating non-point source pollution in fragile lake ecosystems and enhancing agricultural sustainability.

## Supporting information

S1 File Four supplementary tables on soil texture, nutrient classification standards, soil properties, and fertilizer application practices in the Yilong Lake basin, China. (DOCX)

S1 Fig The moisture content magnitude at various soil depths within the same soil texture. (TIF)

S2 Fig Enzyme activity limitations in different soil textures. a, b: Relationships between C-acquiring enzyme activity and N-acquiring or P-acquiring enzyme activity in farmland within the Yilong Lake basin (n = 216), as determined by standardised major axis (type II) regression. The grey dotted line indicates the 1:1 ratio. *** denotes statistical significance at p < 0.001. c, d: The effects of different soil textures on relative C limitation and N/P limitation in farmland.
(TIF)

## References

[1] Jing X, Li L, Chen S, Shi Y, Xu M, Zhang Q. Straw returning on sloping farmland reduces the soil and water loss via surface flow but increases the nitrogen loss via interflow. Agric Ecosyst Environ. 2022;339:108154. doi: 10.1016/j.agee.2022.108154

[2] Zhang Y, Li P, Liu X, Xiao L, Shi P, Zhao B. Effects of farmland conversion on the stoichiometry of carbon, nitrogen, and phosphorus in soil aggregates on the Loess Plateau of China. Geoderma. 2019;351:188–96. doi: 10.1016/j.geoderma.2019.05.037

[3] Xu X, Thornton PE, Post WM. A global analysis of soil microbial biomass carbon, nitrogen and phosphorus in terrestrial ecosystems. Global Ecology and Biogeography. 2012;22(6):737–49. doi: 10.1111/geb.12029

[4] Elser JJ, Sterner RW, Gorokhova E, Fagan WF, Markow TA, Cotner JB. Biological stoichiometry from genes to ecosystems. Ecology Letters. 2000;3(6):540–50. doi: 10.1111/j.1461-0248.2000.00185.x

[5] Sterner RW, Elser JJ. Ecological stoichiometry: the biology of elements from molecules to the biosphere. Princeton University Press. 2002.

[6] Li Y, Zhao Y, Bao X, Xie H, Lü X, Fu Y, et al. Soil total and available C:N:P stoichiometry among different parent material soil profiles in rubber plantations of Hainan Island, China. Geoderma Regional. 2024;36:e00765. doi: 10.1016/j.geodrs.2024.e00765

[7] Yin X, Zhao L, Fang Q, Ding G. Differences in soil physicochemical properties in different-aged *Pinus massoniana* plantations in Southwest China. Forests. 2021;12(8):987. doi: 10.3390/f12080987

[8] Cleveland CC, Liptzin D. C:N:P stoichiometry in soil: is there a “Redfield ratio” for the microbial biomass?. Biogeochemistry. 2007;85(3):235–52. doi: 10.1007/s10533-007-9132-0

[9] Sinsabaugh RL, Hill BH, Follstad Shah JJ. Ecoenzymatic stoichiometry of microbial organic nutrient acquisition in soil and sediment. Nature. 2009;462(7274):795–8. doi: 10.1038/nature08632 20010687

[10] Sinsabaugh RL, Follstad Shah JJ. Ecoenzymatic stoichiometry and ecological theory. Annu Rev Ecol Evol Syst. 2012;43(1):313–43. doi: 10.1146/annurev-ecolsys-071112-124414

[11] Chen G, Yuan J, Wang S, Liang Y, Wang D, Zhu Y, et al. Soil and microbial C:N:P stoichiometries play vital roles in regulating P transformation in agricultural ecosystems: a review. Pedosphere. 2024;34(1):44–51. doi: 10.1016/j.pedsph.2023.06.002

[12] Bhople P, Wall D, Richards K, Clough T, Brennan F, Lanigan G, et al. Soil nutrient stoichiometry impacts on soil organic carbon stocks in long-term phosphorus fertilisation experiments. Geoderma. 2025;463:117538. doi: 10.1016/j.geoderma.2025.117538

[13] Yi C, Zhu J, Chen L, Huang X, Wu R, Zhang H, et al. Speciation of iron and aluminum in relation to phosphorus sorption and supply characteristics of soil aggregates in subtropical forests. Forests. 2023;14(9):1804. doi: 10.3390/f14091804

[14] Six J, Feller C, Denef K, Ogle SM, de Moraes JCS, Albrecht A. Soil organic matter, biota and aggregation in temperate and tropical soils - effects of no-tillage. Agronomie. 2002;22(7–8):755–75. doi: 10.1051/agro:2002043

[15] Dungait JAJ, Hopkins DW, Gregory AS, Whitmore AP. Soil organic matter turnover is governed by accessibility not recalcitrance. Global Change Biology. 2012;18(6):1781–96. doi: 10.1111/j.1365-2486.2012.02665.x

[16] Feller C, Beare MH. Physical control of soil organic matter dynamics in the tropics. Geoderma. 1997;79(1–4):69–116. doi: 10.1016/s0016-7061(97)00039-6

[17] Cameron KC, Di HJ, Moir JL. Nitrogen losses from the soil/plant system: a review. Ann Appl Biol. 2013;162(2):145–73. doi: 10.1111/aab.12014

[18] Plante AF, Conant RT, Stewart CE, Paustian K, Six J. Impact of soil texture on the distribution of soil organic matter in physical and chemical fractions. Soil Science Soc of Amer J. 2006;70(1):287–96. doi: 10.2136/sssaj2004.0363

[19] Riza S, Sekine M, Kanno A, Yamamoto K, Imai T, Higuchi T. Modeling soil landscapes and soil textures using hyperscale terrain attributes. Geoderma. 2021;402:115177. doi: 10.1016/j.geoderma.2021.115177

[20] Wang X, Xue X, Zhang Y, Qin S, You Q, Duan Y, et al. Divergent mechanisms driving nutrient stoichiometry in surface and deep soils of desert ecosystems on the Qinghai–Tibetan plateau. CATENA. 2024;246:108417. doi: 10.1016/j.catena.2024.108417

[21] Su L, Du H, Zeng F, Peng W, Rizwan M, Núñez-Delgado A, et al. Soil and fine roots ecological stoichiometry in different vegetation restoration stages in a karst area, southwest China. J Environ Manage. 2019;252:109694. doi: 10.1016/j.jenvman.2019.109694 31629965

[22] Wang X-G, Lü X-T, Zhang H-Y, Dijkstra FA, Jiang Y-G, Wang X-B, et al. Changes in soil C:N:P stoichiometry along an aridity gradient in drylands of Northern China. Geoderma. 2020;361:114087. doi: 10.1016/j.geoderma.2019.114087

[23] Ma R, Hu F, Liu J, Wang C, Wang Z, Liu G, et al. Shifts in soil nutrient concentrations and C:N:P stoichiometry during long-term natural vegetation restoration. PeerJ. 2020;8:e8382. doi: 10.7717/peerj.8382 32002329 PMC6982410

[24] Xiong J, Shao X, Li N, Yuan H, Liu E, Wu M. Effects of land-use on soil C, N, and P stocks and stoichiometry in coastal wetlands dependent on soil depth and latitude. CATENA. 2024;240:107999. doi: 10.1016/j.catena.2024.107999

[25] Guo X, Jiang Y. Spatial characteristics of ecological stoichiometry and their driving factors in farmland soils in Poyang Lake Plain, Southeast China. J Soils Sediments. 2018;19(1):263–74. doi: 10.1007/s11368-018-2047-7

[26] Chen Q, Li W, Chen A, Min J, Hu W, Wang C, et al. Shallow groundwater table fluctuations promote the accumulation and loss of phosphorus from surface soil to deeper soil in croplands around plateau lakes in Southwest China. J Environ Manage. 2024;366:121744. doi: 10.1016/j.jenvman.2024.121744 38971072

[27] Wang H. Characteristics of runoff nitrogen and phosphorus loss from different farmland types in Dianchi basin. Yunnan University. 2014. https://kns.cnki.net/kcms2/article/abstract?v=3uoqIhG8C475KOm_zrgu4lQARvep2SAk8URRK9V8kZLG_vkiPpTeIdT1m0GuToEdPnULsXVymRnzsFTQ7lQv3e_Mw1SjxYaF&uniplatform=NZKPT

[28] Wu T, Wang S, Su B, Wu H, Wang G. Understanding the water quality change of the Yilong Lake based on comprehensive assessment methods. Ecol Indic. 2021;126:107714. doi: 10.1016/j.ecolind.2021.107714

[29] Liao M, Li K, Sun W, Ni J. Different facets of dry–wet patterns in south-western China over the past 27 000 years. Clim Past. 2021;17(5):2291–303. doi: 10.5194/cp-17-2291-2021

[30] Chen Q, Chen A, Min J, Li L, Hu W, Wang C, et al. Shallow groundwater table fluctuations weaken nitrogen accumulation in the thin layer vadose zone of cropland around plateau lakes, Southwest China. Sci Total Environ. 2024;950:175300. doi: 10.1016/j.scitotenv.2024.175300 39111447

[31] Yuan Z, Wu D, Niu L, Ma X, Li Y, Hillman AL, et al. Contrasting ecosystem responses to climatic events and human activity revealed by a sedimentary record from Lake Yilong, southwestern China. Sci Total Environ. 2021;783:146922. doi: 10.1016/j.scitotenv.2021.146922 33872903

[32] Cui J, Zhang S, Wang X, Xu X, Ai C, Liang G, et al. Enzymatic stoichiometry reveals phosphorus limitation-induced changes in the soil bacterial communities and element cycling: evidence from a long-term field experiment. Geoderma. 2022;426:116124. doi: 10.1016/j.geoderma.2022.116124

[33] Chen L, Liu L, Mao C, Qin S, Wang J, Liu F, et al. Nitrogen availability regulates topsoil carbon dynamics after permafrost thaw by altering microbial metabolic efficiency. Nat Commun. 2018;9(1):3951. doi: 10.1038/s41467-018-06232-y 30262803 PMC6160441

[34] Cui Y, Bing H, Moorhead DL, Delgado-Baquerizo M, Ye L, Yu J, et al. Ecoenzymatic stoichiometry reveals widespread soil phosphorus limitation to microbial metabolism across Chinese forests. Commun Earth Environ. 2022;3(1). doi: 10.1038/s43247-022-00523-5

[35] Du E, Terrer C, Pellegrini AFA, Ahlström A, van Lissa CJ, Zhao X. Global patterns of terrestrial nitrogen and phosphorus limitation. Nature Geoscience. 2020;13(3):221–6. doi: 10.1038/s41561-019-0530-4

[36] Kim DG, Kirschbaum MUF, Eichler-Löbermann B, Gifford RM, Liang LL. The effect of land-use change on soil C, N, P, and their stoichiometries: a global synthesis. Agric Ecosyst Environ. 2023;348:108402. doi: 10.1016/j.agee.2023.108402

[37] Zhang K, Su Y, Yang R. Variation of soil organic carbon, nitrogen, and phosphorus stoichiometry and biogeographic factors across the desert ecosystem of Hexi Corridor, Northwestern China. J Soils Sediments. 2018;19(1):49–57. doi: 10.1007/s11368-018-2007-2

[38] Augusto L, Achat DL, Jonard M, Vidal D, Ringeval B. Soil parent material-a major driver of plant nutrient limitations in terrestrial ecosystems. Glob Chang Biol. 2017;23(9):3808–24. doi: 10.1111/gcb.13691 28317232

[39] Zhang H, Shi L, Lu H, Shao Y, Liu S, Fu S. Drought promotes soil phosphorus transformation and reduces phosphorus bioavailability in a temperate forest. Sci Total Environ. 2020;732:139295. doi: 10.1016/j.scitotenv.2020.139295 32438146

[40] Cheng Y, Li P, Xu G, Li Z, Cheng S, Gao H. Spatial distribution of soil total phosphorus in Yingwugou watershed of the Dan River, China. CATENA. 2016;136:175–81. doi: 10.1016/j.catena.2015.02.015

[41] Zhang S, Chen Y, Lu Y, Guo H, Guo X, Zhou X, et al. C:N:P stoichiometric characteristics and drivers of surface soil in the drylands of China. Environ Earth Sci. 2024;83(9). doi: 10.1007/s12665-024-11489-2

[42] Abrar MM, Xu H, Aziz T, Sun N, Mustafa A, Aslam MW, et al. Carbon, nitrogen, and phosphorus stoichiometry mediate sensitivity of carbon stabilization mechanisms along with surface layers of a Mollisol after long-term fertilization in Northeast China. J Soils Sediments. 2020;21(2):705–23. doi: 10.1007/s11368-020-02825-7

[43] Hamdan J, Bumham CP. The contribution of nutrients from parent material in three deeply weathered soils of Peninsular Malaysia. Geoderma. 1996;74(3–4):219–33. doi: 10.1016/s0016-7061(96)00062-6

[44] Tian H, Chen G, Zhang C, Melillo JM, Hall CAS. Pattern and variation of C:N:P ratios in China’s soils: a synthesis of observational data. Biogeochemistry. 2009;98(1–3):139–51. doi: 10.1007/s10533-009-9382-0

[45] Liu Y, Fu L, Lu X, Yan Y. Characteristics of soil nutrients and their ecological stoichiometry in different land use types in the Nianchu River Basin. Land. 2022;11(7):1001. doi: 10.3390/land11071001

[46] Gao X-L, Li X, Zhao L, Kuzyakov Y. Shrubs magnify soil phosphorus depletion in Tibetan meadows: Conclusions from C:N:P stoichiometry and deep soil profiles. Sci Total Environ. 2021;785:147320. doi: 10.1016/j.scitotenv.2021.147320 33957589

[47] Ding S, Liu Y, Dan SF, Jiao L. Historical changes of sedimentary P-binding forms and their ecological driving mechanism in a typical “grass-algae” eutrophic lake. Water Res. 2021;204:117604. doi: 10.1016/j.watres.2021.117604 34517265

[48] Yang L, Yang Z, Zhong X, Xu C, Lin Y, Fan Y, et al. Decreases in soil P availability are associated with soil organic P declines following forest conversion in subtropical China. CATENA. 2021;205:105459. doi: 10.1016/j.catena.2021.105459

[49] Jiang Y, Rao L, Sun K, Han Y, Guo X. Spatio-temporal distribution of soil nitrogen in Poyang lake ecological economic zone (South-China). Sci Total Environ. 2018;626:235–43. doi: 10.1016/j.scitotenv.2018.01.087 29339265

[50] Yang S, Feng C, Ma Y, Wang W, Huang C, Qi C, et al. Forest Ecology and Management. 2021;494:119298. doi: 10.1016/j.foreco.2021.119298

