# Supplementary material for: Soil texture and depth differentially regulate total and available nutrient stoichiometry in Yilong Lake basin agroecosystem, China
Source: PLoS One. 2026 Oct 29;21(10):e0361813. doi: 10.1371/journal.pone.0361813 (PMC13719017; doi:10.1371/journal.pone.0361813)
Supplement: Supplementary file 1 [file pone.0361813.s001.docx]

**Soil texture and depth differentially regulate total and available nutrient stoichiometry in Yilong Lake basin agroecosystem, China**

**Yongru Lai^1, 2, 3, ¶^****, Luoqi Zhao^1, 2, ¶^, Chenyang Zhang^1, 2, 3^, Zijun Du^1, 2^,**

**Yandan Wang^1, 2, 3^, Tianyi Pu^1, 2^, Change Liu^1, 2, 3^, Changqun Duan^1, 2, 3*^**

^1^ Yunnan Key Laboratory for Plateau Mountain Ecology and Restoration of Degraded Environments, Yunnan University, Kunming, Yunnan, China;

^2^Central Yunnan Field Scientific Station for Restoration of Ecological Function & Yunnan International Joint Research Center of Plateau Lake Ecological Restoration and Watershed Management, Yunnan Think Tank for Ecological Civilization Construction, Yunnan University, Kunming, China;

^3^ Southwestern United Graduate School & Institute of International Rivers and Eco-security, Yunnan University, Kunming, China.

*Corresponding author

Email: [chqduan@ynu.edu.cn](mailto:chqduan@ynu.edu.cn)

^¶^ These authors contributed equally to this work.

S1 Table. Soil Particle Composition and Classification in Various Experimental Areas.

| Soil Sample | clay (%) (<0.002mm) | silt (%)（0.05–0.002mm) | sand (%)（2–0.05mm） | texture type |
| --- | --- | --- | --- | --- |
| 1 | 51.01 | 11.123 | 37.867 | Clay |
| 2 | 47.061 | 14.122 | 38.817 | Clay |
| 3 | 52.494 | 10.494 | 37.012 | Clay |
| 4 | 15.828 | 48.549 | 35.623 | Loam |
| 5 | 23.184 | 49.848 | 26.968 | Loam |
| 6 | 21.183 | 48.791 | 30.026 | Loam |
| 7 | 27.941 | 39.124 | 32.935 | Clay Loam |
| 8 | 29.678 | 43.413 | 26.909 | Clay Loam |
| 9 | 31.109 | 37.565 | 31.326 | Clay Loam |
| 10 | 0.246 | 1.754 | 98 | Sand |
| 11 | 0.001 | 0.157 | 99.842 | Sand |
| 12 | 0.523 | 1.129 | 98.348 | Sand |

S2 Table. China’s second soil census nutrient classification standards.

| Classification | SOC(g·kg^–1^) | TN (g·kg^–1^) | TP (g·kg^–1^) | AN (mg·kg^–1^) | AP (mg·kg^–1^) |
| --- | --- | --- | --- | --- | --- |
| Level I | >23.20 | > 2.00 | > 1.00 | > 150 | > 40 |
| Level II | 17.40–23.30 | 1.50–2.00 | 0.80–1.00 | 120–150 | 20–40 |
| Level III | 11.60–17.40 | 1.00–1.50 | 0.60–0.80 | 90–120 | 10–20 |
| Level IV | 5.80–11.60 | 0.75–1.00 | 0.40–0.60 | 60–90 | 5–10 |
| Level V | 3.48–5.80 | 0.50–0.75 | 0.20–0.40 | 30–60 | 3–5 |
| Level VI | <3.48 | < 0.50 | < 0.20 | < 30 | < 3 |

S3 Table. Soil characteristics as affected by texture soil and sampling depth in agricultural fields in the valley of Yilong Lake, China

|  | pH | C: N: P | DOC: AN: AP |
| --- | --- | --- | --- |
| Texture soil | |  |  |
| Clay | 7.44±0.04c^①^ | 203:53:1 | 900:920:100 |
| Loam | 7.68±0.02b | 56:7:1 | 128:125:100 |
| Clay Loam | 7.81±0.04a | 54:7:1 | 164:122:100 |
| Sand | 7.72±0.03ab | 15:5:1 | 47:30:100 |
|  |  |  |  |
| Depth |  |  |  |
| 0-20cm | 7.58±0.04b | 44:7:1 | 20:17:30 |
| 20-40cm | 7.67±0.03a | 34:6:1 | 35:27:33 |
| 40-60cm | 7.73±0.02a | 48:8:1 | 27:26:25 |
| average | 7.66 | 36:6:1 | 17:38:30 |

Note: Values represent the means and standard errors (Mean ± standard error); different lower case letters indicating significant difference (*p* < 0.05) of pH, SOC, TN, TP, DOC, AN, AP among different soil texture and depths.

S4 Table. Fertiliser application practices by farmers for quarterly crops in farmland within the Yilong Lake Basin, China

| Crop type | Total volume of fertiliser, converted to pure nutrient content | N (kg·ha^–1^) | P_2_O_5_ (kg·ha^–1^) | K_2_O (kg·ha^–1^) |
| --- | --- | --- | --- | --- |
| Grain | 393.14 | 270.66 | 71.30 | 51.18 |
| Fruit | 493.07 | 175.38 | 122.56 | 195.13 |
| Vegetable | 537.45 | 230.13 | 96.52 | 210.8 |
